# INTEREST GROUP SESSION—MENTAL HEALTH PRACTICE AND AGING: INTEGRATION OF SOCIAL DETERMINANTS OF HEALTH IN DEFINING HEALTH BEHAVIORS AND OUTCOMES AMONG DIVERSE OLDER ADULTS

**DOI:** 10.1093/geroni/igz038.1449

**Published:** 2019-11-08

**Authors:** Jacquelyn Minahan, Tamara A Baker

**Affiliations:** University of Kansas, Lawrence, Kansas, United States

## Abstract

Social determinants of health (SDoH) are conditions in which individuals live, learn, work, and play. Specifically, they are influenced by the distribution of resources, money, and power, and have significant implications on health behaviors and outcomes across the life span. Existent data show the influence these indictors may have in the onset and progression of chronic illnesses. However, much of these data focus on the effect of race and health, as social determinants, but fail to adequately address the myriad other factors (e.g., health care, social and community context) that influence the social patterning across the life course. This symposium presents findings from several studies highlighting the nuanced role of SDoH across diverse populations of older adults. Scholars will present findings on the influence that identified determinants, such as social networks, lifestyle behaviors, and gender, have in defining health outcomes across the life course. Minahan presents the relationship between chronic illnesses and depression and compares depressive symptomatology according to disease cluster in a nationally-representative sample of older adults. Atakere discusses determinants of well-being among African American males with chronic illnesses and the challenges associated with this marginalized population. Booker examines spirituality as a mechanism for pain management among older African Americans and presents this as a crucial determinant of health. This symposium will expand on the existing body of literature by emphasizing social and cultural determinants, aside from race, that influence health behaviors and outcomes across the life span.

